# Rasch analysis of the Trypophobia Questionnaire

**DOI:** 10.1186/s13104-018-3245-5

**Published:** 2018-02-14

**Authors:** Shu Imaizumi, Yoshihiko Tanno

**Affiliations:** 1 Graduate School of Arts and Sciences, The University of Tokyo, 3-8-1 Komaba, Meguro, Tokyo, 153-8902 Japan; 2 Japan Society for the Promotion of Science, 5-3-1 Kojimachi, Chiyoda, Tokyo, 102-0083 Japan

**Keywords:** Psychology, Emotion, Disgust, Personality, Individual differences, Psychometric properties, Rasch measurement model

## Abstract

**Objective:**

This study aimed to assess Rasch-based psychometric properties of the Trypophobia Questionnaire measuring proneness to *trypophobia*, which refers to disgust and unpleasantness induced by the observation of clusters of objects (e.g., lotus seed pods).

**Results:**

Rasch analysis was performed on data from 582 healthy Japanese adults. The results suggested that Trypophobia Questionnaire has a unidimensional structure with ordered response categories and sufficient person and item reliabilities, and that it does not have differential item functioning across sexes and age groups, whereas the targeting of the scale leaves room for improvements. When items that did not fit the Rasch model were removed, the shortened version showed slightly improved psychometric properties. However, results were not conclusive in determining whether the full or shortened version is better for practical use. Further assessment and validation are needed.

**Electronic supplementary material:**

The online version of this article (10.1186/s13104-018-3245-5) contains supplementary material, which is available to authorized users.

## Introduction

Trypophobia refers to disgust and/or unpleasantness evoked by images depicting clusters of roughly circular objects that are usually innocuous (e.g., lotus seed pods) [1]. Trypophobic responses can also be somatic, such as itch and nausea [2, 3], affecting both skin conductance [4] and pupil constriction [5]. Trypophobic stimuli may be associated with skin coloration of poisonous animals [1], skin lesions [6, 7], and the observers’ skin disease history [8], and consequently can trigger pathogen-avoidance behaviors as trypophobic responses [3, 8, 9]. Moreover, trypophobic images possess spatial-frequency characteristics likely to induce visuoperceptual discomfort [1, 2, 10]. As these potential factors can vary between individuals, there is substantial inter-individual variability [2]; for instance, 46 of 286 English adults exhibited aversion to a trypophobic image while the others did not [1].

The Trypophobia Questionnaire (TQ) was developed to assess the extent to which respondents can experience subjective and somatic responses expressing disgust and/or unpleasantness induced by trypophobic images [2], and has been employed to elucidate mechanisms of trypophobia [4, 9–12]. Although some studies have confirmed a one-factor structure, reliability, and validity of the TQ [2, 9, 11], there remains room for analyses of its psychometric properties. This study investigated Rasch-based psychometric properties of the TQ. Rasch analysis, in contrast to classical test theory, computes the extent to which the observed responses fit the responses predicted by the Rasch measurement model, and assesses the scale’s unidimensionality and precision in measurement [13, 14].

## Main text

### Methods

#### Participants

We recruited 584 Japanese adults via Lancers [15], a crowdsourcing service. To sample the general population, crowdsourcing was conducted with only the following requirements: participants should be healthy, older than 18 years, and native Japanese speakers. Sample size was based on a guideline indicating that 500 participants are required for precise and robust Rasch analysis [16]. One participant who did not complete the TQ and another who provided invalid responses (i.e., extreme agreement for all items including dummies) were excluded. Data from 582 participants were analyzed [338 females, age 19–81 years, mean = 39.59, standard deviation (SD) = 9.96].

There was no sex or age bias in the sample: a Mann–Whitney test showed no difference in age between sexes (*U* = 44,375, *p* = 0.117, *ρ*_rb_ = 0.076; ages in males and females were not normally distributed, Shapiro–Wilk *W*s < 0.98, *p*s < 0.001). Participants younger than or equal to the median age of 39 years were comparable to those older in terms of male/female ratio (*χ*^2^(1) = 0.05, *p* = 0.833, *φ* = 0.009).

#### Procedures

Participants were directed to a survey website generated by Qualtrics [17] via their own computers. They reported their sex and age, completed the TQ, and were paid 162 Japanese yen (approximately 1.4 US dollars).

The TQ is a one-factor structure questionnaire including 17 items (Table 1) to assess proneness to subjective and somatic responses induced by trypophobic images and two dummy items irrelevant to the construct of interest (“Want to laugh,” “Feel at peace”) [2]. We employed the Japanese version, which has been validated for use in an adult sample [18]. Participants observed two trypophobic images (lotus seed pods, honeycombs) as in the original [2], and rated their agreement with each item on a 5-point scale ranging from 1 (“Not at all”) to 5 (“Extremely”). The scale score was the summed item scores, excluding dummy items.

**Table 1:** The Trypophobia Questionnaire and its Rasch-based psychometric properties

	Location (standard error)	Infit mean-square (Zstd)	Outfit mean-square (Zstd)	First contrast loading	Second contrast loading	DIF contrast for sex	DIF contrast for age
Feel freaked out	− 0.50 (0.06)	1.07 (1.10)	1.08 (1.00)	0.55	− 0.17	0.14	0.00
Feel aversion, disgust or repulsion	− 1.75 (0.05)	0.88 (− 2.10)	0.88 (− 1.80)	0.61	0.41	0.16	0.08
Feel uncomfortable or uneasy	− 1.68 (0.05)	0.73 (− 4.80)	0.80 (− 3.10)	0.63	0.23	0.11	0.10
Feel like panicking or screaming	0.33 (0.07)	0.98 (− 0.20)	0.83 (− 1.70)	0.24	− 0.45	0.12	0.08
Feel anxious, full of dread or fearful	− 0.52 (0.06)	0.87 (− 2.00)	0.84 (− 2.10)	0.46	− 0.31	0.10	0.00
Feel sick or nauseous	0.02 (0.06)	0.87 (− 1.90)	0.74 (− 3.10)	− 0.13	− 0.24	0.00	0.06
Feel nervous (e.g., heart pounding, butterflies in stomach, sweating, stomachache, etc.)	0.05 (0.06)	0.92 (− 1.10)	0.87 (− 1.40)	− 0.09	− 0.21	0.17	0.06
Feel like going crazy	0.00 (0.06)	0.82 (− 2.60)	0.79 (− 2.40)	− 0.14	− 0.27	0.00	0.22
Have an urge to destroy the holes	0.97 (0.08)	2.16 (9.90)	2.32 (7.10)	− 0.06	− 0.22	0.51	0.33
Feel itchiness	0.24 (0.06)	1.56 (6.60)	1.40 (3.60)	− 0.16	0.18	0.22	0.20
Feel skin crawl	− 0.90 (0.05)	0.86 (− 2.30)	0.82 (− 2.70)	− 0.20	0.66	0.17	0.09
Have goosebumps	− 0.83 (0.05)	0.96 (− 0.70)	0.93 (− 0.90)	− 0.40	0.62	0.08	0.08
Feel like crying	1.96 (0.10)	1.57 (4.50)	0.89 (− 0.50)	− 0.37	− 0.37	0.13	0.19
Vomit	1.40 (0.08)	1.08 (0.90)	0.77 (− 1.40)	− 0.41	− 0.24	0.37	0.07
Get chills	− 0.26 (0.06)	1.14 (2.10)	1.01 (0.10)	− 0.43	0.35	0.05	0.10
Have trouble breathing	0.63 (0.07)	0.97 (− 0.40)	0.86 (− 1.20)	− 0.27	− 0.37	0.07	0.21
Shiver	0.83 (0.07)	0.98 (− 0.20)	0.72 (− 2.40)	− 0.47	− 0.16	0.22	0.11

Differential item functioning contrasts were reported in absolute values. Zstd, z-standardized statistic; DIF, differential item functioning

#### Data analysis

Descriptive statistics of the TQ except for dummy items and its relationships with sex and age were analyzed using JASP 0.8.5.1 [19]. Rasch analysis was performed using Winsteps 4.0.1 [20]. As all items shared the same polytomous response structure [21], the rating scale model was employed [22]. The procedures of Rasch analysis were based on recent guidelines [21, 23].

Ordering of thresholds between five response categories of the TQ was assessed. Thresholds refer to points at which two adjacent curves cross. Disordering implies underused and/or indistinguishable categories.

Unidimensionality (i.e., to what extent the scale assesses single construct) was assessed by principal component analysis (PCA) of the residuals based on the amount of raw variance explained by the measure and the eigenvalue of unexplained variance in the first contrast (i.e., latent dimension). As in the previous studies, we also reported factor analysis for descriptive purposes.

Infit and outfit mean-squares for each item were the indices of the fit to Rasch unidimensional model. Infit mean-square is based on the Chi square statistic weighted using model variance and sensitive to inliers. Outfit mean-square is based on the conventional Chi square statistic and sensitive to outliers. Because mean-squares indicate the amount of distortion of the measurement system and their expected values are close to 1.00, values less than 1.00 indicate overfit and those greater than 1.00 indicate underfit to the model. We also reported infit and outfit *z*-standardized statistics (i.e., standardized *t*-statistics with infinite degrees of freedom), which indicate statistical significance of mean-squares.

Rasch measure, which was computed for each person and item and expressed in logits, indicates the location on the unidimensional latent variable. We assessed *targeting*, which is the difference between mean person and item location measures and indicates how well item difficulties match individuals’ abilities. Differential item functioning (DIF) can be assessed by subgroup differentials of the Rasch item measure, indicating whether a subgroup of a sample scores on an item different from another subgroup. We assessed DIF across two subgroups: sex (male versus female) and age (younger or equal to versus older than the median of 39 years).

Person and item reliabilities (i.e., reproducibility) based on the Rasch model were analyzed. High person or item reliability indicates high probability that persons or items with high estimated measures indeed show higher measures than do persons or items with low estimated measures. Specifically, person reliability reflects reproducibility of person ordering that can be expected if the same sample responded to another set of items measuring the same construct, and item reliability reflects reproducibility of items’ hierarchy and/or given item scores if another sample responded to the same items [24]. We also reported internal consistency (i.e., Cronbach’s alpha) for descriptive purposes.

### Results and discussion

#### Descriptive statistics

The mean TQ score was 32.02 [SD = 13.71; range 17–85; skewness (standard error) = 1.10 (0.10); kurtosis = 0.70 (0.20)]. TQ scores in total, male, and female samples were not normally distributed (*W*s < 0.90, *p*s < 0.001). While we found no sex difference in TQ score (Mean_male_ = 31.44, SD_male_ = 12.66, Mean_female_ = 32.44, SD_female_ = 14.44, *U* = 40,596, *p* = 0.749, *ρ*_rb_ = − 0.016), age negatively but weakly correlated with TQ score (*ρ* = − 0.227, *p* < 0.001). These (null) effects of sex and age on TQ were consistent with previous studies [9, 18]. As it is outside the scope of this study, relief from trypophobia with age should be investigated by future research.

#### Rasch analysis

**Fig. 1:**
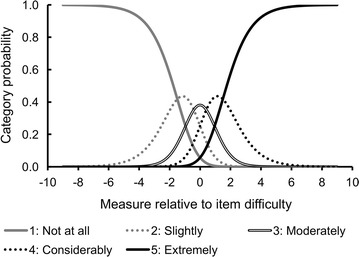
Category probability curves for the Trypophobia Questionnaire. The curves show ordered thresholds between five response categories

We confirmed continuous ordering of thresholds of the five response categories (Fig. 1); their average measures were − 2.96, − 1.47, − 0.49, 0.40, and 1.37, respectively [21]. This suggested that all response categories were distinguished and evenly used by participants.

Rasch-based PCA showed that the measures explained 64.1% of the raw variance, which was above the criterion of 50.0% for unidimensionality of the scale [23]. The eigenvalues of the unexplained variance in the first and second PCA contrasts (i.e., latent dimensions) were 2.38 and 2.10, respectively, which exceeded the cutoff of 2.00 [23]. These suggested that the TQ possesses unidimensionality, but also that there may be other latent dimensions in the residuals. We thus examined the presence of multidimensionality in terms of the correlation between item clusters within each contrast [21]. The items were separated into three item clusters based on each of the first and second contrast loadings (Table 1). The correlation between item clusters was reported as disattenuated Pearson correlation coefficient, which removed the standard error of measurement for each item cluster. When the coefficient approaches 1.000, a pair of item clusters measures the same construct [21]. We indeed found that the coefficients were very high: 0.928–1.000 for the first contrast and 0.988–1.000 for the second. This suggests that the item clusters defined by two latent dimensions (i.e., contrast) indeed measure the same construct, supporting the unidimensionality of the TQ. Factor analysis also confirmed its one-factor structure, consistent with previous studies [2, 11, 18] (see Additional file 1).

Most items were well fitted to the unidimensional model; infit and outfit mean-squares were between 0.73 and 1.14, within a criterion range (i.e., 0.70–1.30), except for the items “Have an urge to destroy the holes,” “Feel itchiness,” and “Feel like crying,” whose infit and/or outfit mean-squares were above 1.30 (Table 1). These can be interpreted as low-quality fit to the scale [23]. Therefore, these three items might be candidates to be removed from the TQ (see next section).

**Fig. 2:**
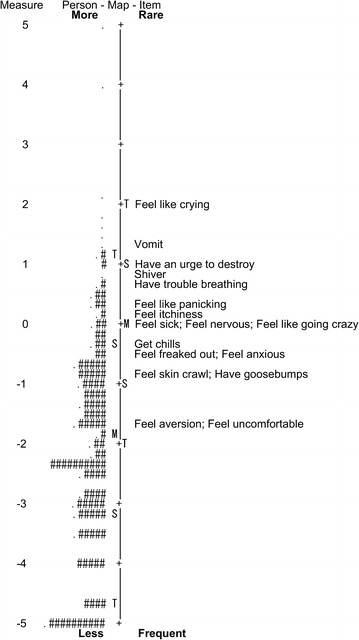
Wright’s person-item map of the Trypophobia Questionnaire. Person locations are on the left column, item locations are on the right. Each period represents one to four participants, and each hash represents five. The item names on the right column were abbreviated for brevity. M, mean; S, standard deviation from the mean; T, two standard deviations from the mean

The Rasch person and item location measures are displayed in a Wright map (Fig. 2). The targeting index of 2.13 [i.e., item measure: mean (SD) = 0.00 (0.97); person measure = − 2.13 (1.89)] exceeded the cutoff of 2.00, suggesting that the TQ has a low level of matching between item difficulty and person ability [23]. This might be because a minority of individuals experience trypophobia, given that a previous study reported that 16.1% of adults exhibited aversion to a trypophobic image [1]. All items showed insignificant DIFs for sex (i.e., differentials of item measures less than 0.37, not exceeding a cutoff of 0.50; Table 1), except for the item “Have an urge to destroy the holes,” which showed a DIF of 0.51 and was unfit for the model (see above). Moreover, all items showed an insignificant DIF for age, at less than 0.33. While zero-order correlations suggested a weak correlation between TQ and age in the present and previous [18] studies, the present Rasch analysis suggested that the TQ indeed possesses unproblematic DIF and remains stable regardless of sex and age [23].

The person reliability of 0.86 and item reliability of 0.99 were above the criteria for sufficiency of 0.80 and 0.90, respectively [21], suggesting that the TQ has sufficient reproducibility of respondent classification and item hierarchy. Furthermore, the Cronbach’s alpha of 0.95 was sufficiently high and comparable to the previous studies [2, 9, 11, 18], demonstrating good internal consistency of the TQ.

In sum, the TQ possesses a unidimensional structure with ordered response categories measuring a single construct (i.e., proneness to trypophobia) and has sufficient reproducibility, although the targeting leaves room for improvement. Nevertheless, three items did not fit well to the unidimensional structure. A shortened version without these items might improve psychometric properties.

#### Follow-up without unfit items

We performed follow-up Rasch analysis without the three unfit items (see Additional file 1 for details). Infit and outfit mean-squares for the 14-item version of TQ were within the criterion range (infit: 0.78–1.29; outfit: 0.81–1.22), demonstrating a better fit to the model, as expected. Response categories ordered well again. PCA revealed that 67.1% of the raw variance was explained by measures, but the eigenvalue of the first contrast was 2.33, exceeding the cutoff. Nevertheless, item clusters defined by the first contrast loading were highly correlated (i.e., disattenuated correlation coefficients of 0.849–1.000), suggesting unidimensionality comparable to the full version of the TQ. Targeting of 2.11 indicated low quality, comparable to the full version. The DIFs were inconsequential, as all differentials of item measures across sex and age subgroups were less than 0.46. The person reliability of 0.87 and item reliability of 0.99 were sufficiently high, comparable to the full version.

Although psychometric properties of the shortened TQ improved slightly, the full and shortened versions had comparable qualities according to the criteria [21, 23]. To determine whether the TQ should be formally shortened, further studies should compare the validity of the full and shortened versions by examining behaviors [2, 18] and other psychological constructs (e.g., anxiety [2, 11], disgust sensitivity [3, 9]).

### Conclusions

Rasch analysis suggested that the TQ has a unidimensional structure with ordered response categories and sufficient person and item reproducibility, although the targeting leaves room for improvement. Although inconclusive, a revised TQ without unfit items might improve its psychometric properties, but further comparative studies and validations are required.

## Limitations

Rasch-based psychometric properties of the TQ were shown using its Japanese version and online sampling. To generalize our findings, future studies should replicate the results using the English version and paper-and-pencil sampling.

## Additional files

**Additional file 1.** Supplemental results for the full and short versions of the Trypophobia Questionnaire. This file describes factor analysis on the full version of the Trypophobia Questionnaire, and follow-up analyses on the shortened 14-item version of the scale, including descriptive statistics, Rasch analysis, and factor analysis.

**Additional file 2.** Raw data from the survey. Dataset includes demographic data and responses to the Trypophobia Questionnaire from 582 healthy native Japanese speakers who were recruited via online.

## Abbreviations

DIF: differential item functioning
PCA: principal component analysis
SD: standard deviation
TQ: Trypophobia Questionnaire
