# Supplementary material for: Rasch analysis of the Trypophobia Questionnaire
Source: BMC Res Notes. 2018 Feb 14;11:128. doi: 10.1186/s13104-018-3245-5 (PMC5813320; doi:10.1186/s13104-018-3245-5)
Supplement: Supplementary file 1 — Additional file 1. Supplemental results for the full and short versions of the Trypophobia Questionnaire. This file describes factor analysis on the full version of the Trypophobia Questionnaire, and follow-up analyses on the shortened 14-item version of the scale, including descriptive statistics, Rasch analysis, and factor analysis. [file 13104_2018_3245_MOESM1_ESM.pdf]

Additional file 1: Supplemental results for the full and short versions of the Trypophobia Questionnaire

Full version

To replicate previous studies [1, 2], factor analysis using the maximum-likelihood method with promax rotation was performed on the 17 items of the Japanese version [1] of the Trypophobia Questionnaire (TQ) [3] using SPSS 24.0 (IBM, Armonk, New York). Data from 582 adults were analyzed. As a result, the Kaiser–Meyer–Olkin index of sampling validity was 0.954 and Bartlett's test for sphericity was significant ($\chi^2(136) = 7399.78$, $p < 0.001$), suggesting that this analysis was valid. As expected, the first factor had an eigenvalue of 9.62 and explained 56.56% of the variance, while the second factor had an eigenvalue of 1.26 and explained 7.41% of the variance. Given the large difference between eigenvalues of the two factors, we interpreted the TQ as a one-factor structure scale, consistent with previous studies [1-3]. Factor loadings ranged from 0.418 to 0.834 (Table S1), again implying that all items loaded on a single factor, thought to be a proneness to trypophobia.

Table S1. One-factor structure of the Trypophobia Questionnaire.

	Full version		14-item version	
	Factor loading	Communality	Factor loading	Communality
Feel freaked out	0.732	0.548	0.734	0.548
Feel aversion, disgust or repulsion	0.827	0.843	0.837	0.847
Feel uncomfortable or uneasy	0.834	0.814	0.843	0.816
Feel like panicking or screaming	0.755	0.582	0.755	0.586
Feel anxious, full of dread or fearful	0.787	0.624	0.789	0.625
Feel sick or nauseous	0.808	0.685	0.806	0.696
Feel nervous (e.g., heart pounding, butterflies in stomach, sweating, stomachache, etc.)	0.787	0.639	0.785	0.646
Feel like going crazy	0.795	0.659	0.794	0.669
Have an urge to destroy the holes	0.418	0.200	–	–
Feel itchiness	0.634	0.413	–	–
Feel skin crawl	0.822	0.678	0.821	0.674
Have goosebumps	0.795	0.633	0.792	0.631
Feel like crying	0.493	0.384	–	–
Vomit	0.641	0.558	0.629	0.546
Get chills	0.747	0.576	0.742	0.575
Have trouble breathing	0.724	0.626	0.713	0.609
Shiver	0.720	0.649	0.711	0.642

Shortened 14-item version

For descriptive purposes, in the same manner as the full version, we analyzed responses from the same 582

adults to the 14-item version of the TQ, excluding unfit items "Have an urge to destroy the holes," "Feel itchiness," and "Feel like crying"). For details about interpreting the results, see the Main Text.

The mean TQ score was 27.65 [standard deviation (SD) = 12.26; range 14–70; skewness (standard error) = 1.05 (0.10); kurtosis = 0.50 (0.20)]. The TQ scores in the total, male, and female samples were not normally distributed (Ws < 0.91, ps < 0.001). While there was no sex difference in TQ score ($\text{Mean}_{\text{male}}$ = 27.12, SD_{male} = 11.32, $\text{Mean}_{\text{female}}$ = 28.02, $\text{SD}_{\text{female}}$ = 12.90, U = 40665, p = 0.775, ρ_{rb} = -0.014), age weakly correlated with TQ score (ρ = -0.221, p < 0.001). These (null) effects of sex and age were consistent with the full version.

Ordering of thresholds of the five category probability curves (Figure S1) and average measures for the five categories (i.e., 1 to 5) of -3.13, -1.58, -0.47, 0.59, and 1.88, respectively, suggested that all response categories were distinguished and evenly used, consistent with the full version.

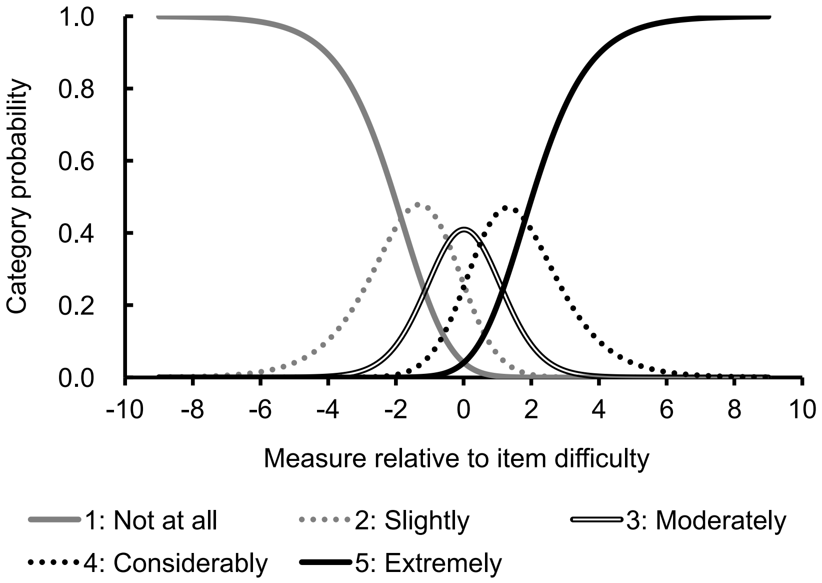

Figure S1. Category probability curves for the 14-item version of the Trypophobia Questionnaire.

As expected, 14 items were well fitted to the unidimensional model; infit and outfit mean-squares ranged from 0.78 to 1.29 (Table S2), within the criterion range of 0.70–1.30. Similar to the full version, principal component analysis showed that the measures explained 67.1% of the raw variance, and the eigenvalues of the unexplained variance in the first and second contrasts were 2.33 and 1.99, respectively. These suggested that the TQ has unidimensionality, but also other latent dimensions in the residuals. To examine potential multidimensionality in the scale, we analyzed the disattenuated Pearson correlation between three item clusters based on each of the first and second contrast loadings (Table S2). The coefficients were very high: 0.849–1.000 for the first contrast and 0.980–1.000 for the second. This suggests that the item clusters defined by two latent dimensions measure the same construct, suggesting unidimensionality. For descriptive purposes, factor analysis—the same as in the full version—was performed on the short version. Kaiser–Meyer–Olkin index of 0.950 and significance in Bartlett's test ($\chi^2(91)$ = 6658.18, p < 0.001) supported the validity of factor analysis. The first factor had an eigenvalue of 8.30 and explained 59.26% of variance, while the second had an eigenvalue of 0.81 and explained 5.81% of the variance. Factor loadings for all items were above 0.629 (Table S1), again implying that all items

loaded on a single factor. The short version also had a unidimensional one-factor structure.

Table S2. The 14-item version of the Trypophobia Questionnaire and its psychometric properties. Differential item functioning (DIF) contrasts are reported in absolute values. Zstd: z-standardized statistic.

	Location (standard error)	Infit mean-square (Zstd)	Outfit mean-square (Zstd)	First contrast loading	Second contrast loading	DIF contrast for sex	DIF contrast for age
Feel freaked out	-0.31 (0.06)	1.19 (2.80)	1.22 (2.70)	-0.55	-0.05	0.18	0.00
Feel aversion, disgust or repulsion	-1.72 (0.06)	0.92 (-1.30)	0.90 (-1.50)	-0.51	0.54	0.18	0.08
Feel uncomfortable or uneasy	-1.64 (0.06)	0.78 (-3.90)	0.84 (-2.50)	-0.55	0.37	0.12	0.10
Feel like panicking or screaming	0.63 (0.07)	1.08 (1.10)	0.95 (-0.40)	-0.30	-0.41	0.11	0.10
Feel anxious, full of dread or fearful	-0.34 (0.06)	0.96 (-0.60)	0.93 (-0.90)	-0.50	-0.22	0.13	0.00
Feel sick or nauseous	0.28 (0.07)	0.96 (-0.50)	0.81 (-2.20)	0.12	-0.38	0.00	0.06
Feel nervous (e.g., heart pounding, butterflies in stomach, sweating, stomachache, etc.)	0.32 (0.07)	1.01 (0.20)	0.96 (-0.40)	0.07	-0.31	0.18	0.07
Feel like going crazy	0.26 (0.07)	0.90 (-1.40)	0.86 (-1.50)	0.13	-0.36	0.06	0.25
Feel skin crawl	-0.76 (0.06)	0.95 (-0.80)	0.90 (-1.50)	0.39	0.59	0.19	0.09
Have goosebumps	-0.69 (0.06)	1.07 (1.00)	1.01 (0.20)	0.59	0.48	0.08	0.09
Vomit	1.84 (0.09)	1.23 (2.30)	0.91 (-0.50)	0.35	-0.34	0.46	0.12
Get chills	-0.04 (0.06)	1.29 (4.00)	1.16 (1.80)	0.51	0.22	0.07	0.11
Have trouble breathing	0.97 (0.07)	1.12 (1.50)	0.99 (0.00)	0.18	-0.42	0.10	0.23
Shiver	1.20 (0.08)	1.10 (1.20)	0.81 (-1.50)	0.43	-0.24	0.27	0.10

Rasch person and item location measures are displayed in a person-item map (Figure S2). The targeting of 2.11 [mean item measure (SD) = 0.00 (0.98); mean person measure = -2.11 (2.09)] exceeded the cutoff of 2.00, suggesting that the short version had a low quality of matching between item difficulty and person ability, comparable to the full version. All items showed inconsequential levels of differential item functioning for sex and age (i.e., differentials of item measures less than 0.46 below the cutoff of 0.50; Table S2). The person reliability of 0.87 and item reliability of 0.99 were above the criteria for sufficiency of 0.80 and 0.90, respectively. The classical internal consistency index Cronbach's alpha was 0.95 and sufficiently high. The short version of the TQ showed sufficient reliability, comparable to the full version

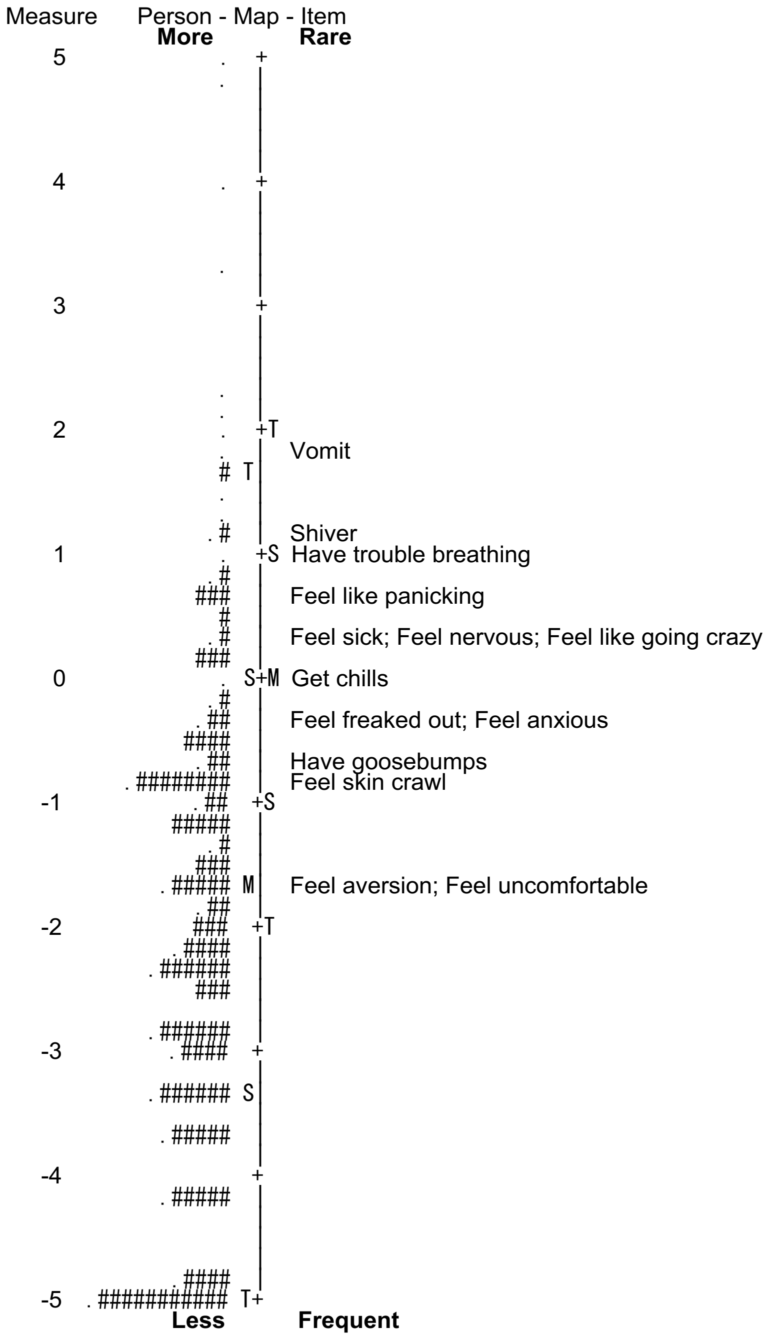

Figure S2. Person-item map of the 14-item version of the Trypophobia Questionnaire. Each period represents one to four participants, and each hash represents five. The item names on the right column are abbreviated for brevity. M: mean; S: standard deviation from the mean; T: two standard deviations from the mean.

Supplemental references

1. Imaizumi S, Furuno M, Hibino H, Koyama S. Development of the Japanese version of Trypophobia Questionnaire. Jpn J Pers. 2016;25:171-3.
2. Chaya K, Xue Y, Uto Y, Yao Q, Yamada Y. Fear of eyes: triadic relation among social anxiety, trypophobia, and discomfort for eye cluster. PeerJ. 2016;4:e1942.
3. Le AT, Cole GG, Wilkins AJ. Assessment of trypophobia and an analysis of its visual precipitation. Q J Exp Psychol (Hove). 2015;68:2304-22.
